# *hsa-miR-423* rs6505162 Is Associated with The Increased Risk of Breast Cancer in Isfahan Central Province of Iran

**DOI:** 10.22074/cellj.2020.7011

**Published:** 2020-09-08

**Authors:** Nadia Pourmoshir, Gholamreza Motalleb, Sadeq Vallian

**Affiliations:** 1 Division of Cell and Molecular Biology, Department of Biology, Faculty of Science, University of Zabol, Zabol, Iran; 2 Division of Genetics, Department of Cellular and Molecular Biology and Microbiology, Faculty of Science and Technology, University of Isfahan, Isfahan, Iran

**Keywords:** Breast Cancer, *hsa-miR-423*, microRNA

## Abstract

**Objective:**

Thirteen million cancer deaths and 21.7 million new cancer cases are expected in the world by 2030. Breast cancer is considered as the main cause of cancer mortality in women aged 20-59 years. microRNAs (miRNAs) regulate gene expression at the post-transcriptional level and they are highly expressed in malignancies, including breast cancer. The role of miRNAs in the pathogenesis of breast cancer is not fully understood. In the present study, for the first time, the impact of *hsa-miR-423* rs6505162 on breast cancer risk was investigated in the central province of Iran, Isfahan.

**Materials and Methods:**

This case-control study was conducted on 153 clinicopathological proven breast cancer patients and 153 sex-matched healthy women with no history of any cancer type and relative patients. The patients and controls were genotyped and association of their clinical characteristics with *hsa-miR-423* rs6505162 genotype was analyzed.

**Results:**

The findings indicated that CC genotype of *hsa-miR-423* rs6505162 was associated with the increased risk of breast cancer [odds ratio (OR)=2.37, 95% confidence interval (CI)=1.29-4.35 and P=0.0023, CC vs. AA].

**Conclusion:**

The data suggested that *hsa-miR-423* rs6505162 could be considered as a novel risk factor in breast cancer pathogenesis in Isfahan province of Iran.

## Introduction

The rate of cancers is increasing day by day in the worldwide. Approximately, 30000 Iranians lose their life due to cancer each year (1). Breast cancer is the most frequent carcinoma and the second leading cause of cancer mortality among women in less-developed countries (2). Multiple studies have made progress in many fields of breast cancer investigations to understand the etiology of its carcinogenesis. However, the precise mechanisms of breast cancer carcinogenesis remain largely unknown (3, 4). It is well accepted that environmental and genetic factors are two main groups of risk factors for breast cancer. Several studies have revealed that the sophisticated synergy of genetics, environmental exposures, hormones and diet behaviors may predispose to breast cancer (5). Early detection of breast cancer primary tumors is very important, because it can increment the chance of an effective cure in patients with early stage of the disease. However, majority of the cancers at an early stage are difficult to detect. Therefore, novel biomarkers for identifying high-risk populations as well as new strategies for early detection are urgently required (6, 7).

microRNAs (miRNAs) are an abundant class of ancient, noncoding and small single-stranded molecules (20- 22 nucleotides) that participate in transcriptional and translational regulations of their target genes (8). Since miRNAs can regulate several target transcripts, they have been identified to have vital roles in normal biological processes, such as cell differentiation, proliferation, immune system regulation, hematopoiesis and apoptosis through different gene regulation networks (9). Elevated or decreased expression of specific miRNAs has been reported to be implicated in down- or up-regulation of the miRNA putative targets, which could give rise to deregulation of the pathways in which those targets are involved. Aberrant expression of miRNA can occur in the multiple processes of carcinogenesis, including cell growth, apoptosis, differentiation, invasion and angiogenesis of solid cancers, including the breast (10, 11). The wide role of miRNAs opens up a new avenue of investigation for molecular mechanisms of miRNAs in cancer pathogenesis. In this framework, miRNA signatures have obviously been distinguished for certain types of malignancy, in which they can act as either tumor suppressors or oncogenes (12). miRNA expression can be altered by various mechanisms, such as chromosomal instability, epigenetic changes, genomic mutations, defects in the mechanisms of miRNA biosynthesis and single nucleotide polymorphisms (SNPs) in their coding genes (13).

In modern genetics, phenotypic variations for traits with medical importance are of special interests. Some variations are due to SNP in miRNAs, named as MirSNPs, which represent a new class of potential biomarkers. These markers have attracted increasing interests due to their potential role in the development of various types of cancer (14). In fact, MirSNPs could be used as new biomarkers for molecular diagnosis of genetic diseases and cancer (15). Many reports indicated that MirSNPs could play functional roles in different ways such as altering the transcription of the primary target genes, affecting premiRNA/ pre-miRNA processing or by exerting effects on miRNA-mRNA interplays (16, 17). Therefore, MirSNPs seem to be ideal biomarkers for constructing genetic maps and categorizing the direct functional and effective variants correlated with common and even genetically complex diseases like cancer.

Various reports from the studies performed on a number of populations with breast cancer patients have demonstrated that SNPs in the precursor of miRNAs (pre-miRNAs), exclusively miR-423 A/C polymorphism, could affect maturation or expression of the respective mature miRNA (18-20).

To date, there is no report on the impact of *hsa-mirR-423* rs6505162 variants on breast cancer risk in the Iranian population. Therefore, the current study was carried out to find the possible association between *hsa-mirR-423* rs6505162 variants polymorphisms and susceptibility to breast cancer in the Isfahan central province of Iran.

## Materials and Methods

### Patients and controls

This study was qualified by meeting the following criteria: i. Designed as case-control, ii. Assessed the association between Hsa-miR-423 rs6505162 polymorphism and breast cancer risk, iii. Provided adequate numbers of genotype distribution in case and control groups, in order to calculate the odd ratios (OR) and its 95% confidence interval (CI).

In the present case-control study, 153 geneticallyunrelated females with breast cancer from the Omid Hospital (Isfahan, Iran) were investigated between 2011 and 2014. As control, the same numbers of healthy independent females were included in the study. The study design and recruitment procedures were described previously (21). The project was approved by the Scientific and Ethical Advisory Group of the University of Zabol (code: 7611) and University of Isfahan (code: 790205), Iran. All control and patient samples were obtained from Isfahan province, which may indicate approximate similarity of healthy and patient population in terms of environmental factors. The informed consent was obtained from all individuals participated in this study. As indicated, the patients were categorized in two groups, including superficial (levels 0, I and II) and invasive (levels III and IV) based on their tum or grade.

**Table 1:** Sequence of the primers designed and used for *miR-423* genotyping (rs6505162)


Primers	Primer sequence (5´-3´)	Amplicon size (bp)

hsa-mir-423rs6505162 A>C		
Forward outer	TTTAAATGCGCTGGAAGTGAAG	410
Reverse outer	CCTATATGCCTACCCTTTTTCTGTG	410
Forward normal	CCCTCAGTCTTGCTTCCCAA	200
Forward mutant	CCCTCAGTCTTGCTTCCCAC	200


### Single nucleotide polymorphisms selection and genotyping

The *hsa-miR-423* rs6505162 polymorphism was selected by considering the following criteria: i. the minor allele frequency (MAF) of selected SNP was < 5% (0.4978), ii. It was functional (22). The International HapMap Project (http://www.hapmap.org), dbSNP (http://www.ncbi.nlm. nih.gov/projects/SNP/) and Mirbase (http://microrna. sanger.ac.uk) were used for evaluation of MAF criteria. F-SNP (http://compbio.cs.queensu.ca/F-SNP) database was used to prioritize the SNPs with potential pathological effect on human health. Genotyping was performed on total genomic DNA. Genomic DNA was extracted from the peripheral blood leukocytes by a standard salting out procedure (23). Genotyping of *hsa-miR-423* rs6505162 polymorphism was carried out by ARMS-PCR using newly designed specific primers (24, 25). Primers were designed using Oligo software and NCBI BLAST search engine as shown in Table 2. The 3´-terminus of each primer was modified to match the corresponding polymorphism in *hsa-miR-423.* Moreover, an artificial mismatch at the antepenultimate base was included in the allele-specific primers to improve the primers/template specificity (26). PCR reactions were performed in two separate tubes in 25μl total volume. The reaction mixture was composed of 1μg DNA, 3 mM MgCl_2_, 0.4 mM dNTPs, 1x PCR Buffer, 1 U Taq DNA polymerase and 0.5μM of each forward and reverse primers. Initial denaturation was accomplished at 94˚C for 5 minutes, followed by 30 cycles including denaturation at 94˚C for 1 minute, annealing temperature at 57˚C for 1 minute, extension at 72˚C for 1 minute, followed by a final extension at 72˚C for 10 minutes. The amplification products were separated in 2% agarose gel and visualized under UV light.

### Statistical analysis

Association of the genotypes with breast cancer was assessed by computing the OR and 95% CI. The data were statistically evaluated using Simple Interactive Statistical Analysis (SISA) software (two by two tables available from http://www.quantitativeskills. com/sisa/). The genotypic associations were examined using SNPstats, (http://bioinfo.iconcologia. net/SNPstats_web). The results were considered statistically significant at P≤0.05.

The Pearson’s chi-square χ2 test was used to evaluate the Hardy-Weinberg equilibrium by considering statistical significance at a P≥0.05 (27). Population studies were carried out by using GENEPOP website (http://genepop.curtin.edu.au) and the polymorphism information content (PIC) was computed by PIC calculator website (https://www.liverpool. ac.uk/~kempsj/pic.html).

### Determination of the best fitting pattern of inheritance

The patterns of inheritance were determined by Akaike information criterion (AIC) and Bayesian information criterion (BIC) (28).

## Results

Clinical and pathological characteristics of the breast ancer patients were summarized in table 2.

**Table 2:** Clinicopathological characteristics of the patients with breast cancer


Characteristics	Number	Percentage
Age (Y), mean ± SD		

≤50	93	60.8
>50	60	39.2
Tumor stage		
Invasive	100	65.4
Superficial	53	34.6
Estrogen receptor		
Positive	94	61.4
Negative	59	38.6
Progesterone receptor		
Positive	90	58.8
Negative	63	41.2


**Fig.1:**
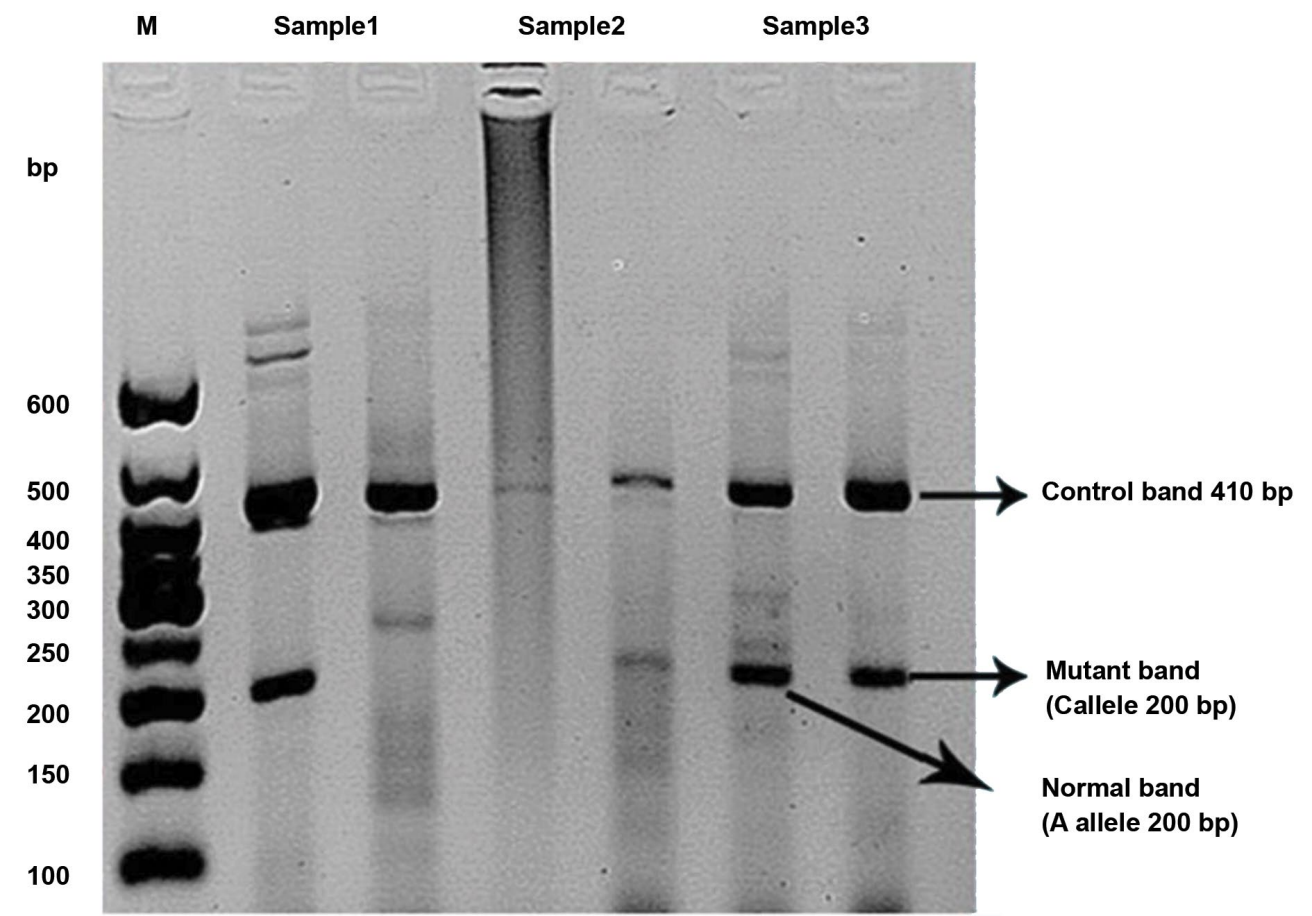
Genotyping of *hsa-miR-423* rs6505162 A>C polymorphism. The results of representative amplified products from ARMS-PCR reactions are shown. The PCR products were run on 2% agarose gel and the *hsa-miR-423* rs6505162 A>C genotypes were detected. Each sample consists of two lines: the first line refers to the allele from normal primers (A allele) and second line refers to the allele of mutant primers (C allele). Therefore, as illustrated, sample 1 (line 1-2) represents a normal homozygous individual (AA), sample 2 (lines 3-4) represents a mutant homozygous individual (CC) and sample 3 (lines 5-6) represents a heterozygous individual (AC). M represents DNA size marker.

Genotyping was performed for *hsa-miR-423* rs6505162 in 153 breast cancer patients (age with mean ± SD of 47.14 ± 10.45 years old] and 153 control individuals [age with mean ± SD of 45.4 ± 12.3 years old]. The results of representative polymerase chain reaction (PCR) products from ARMS-PCR reactions were shown in Figure 1. Data showed that the Pearson Chi-Square significance value was 0.00071 (P<0.05). Analysis of the association of genotyping data with the risk of breast cancer using SNPstats, showed a clear association of *hsa-miR-423* rs6505162 in codominance, recessive and Log-additive patterns with breast cancer. These data showed the presence of possible relationship between *hsa-miR-423* rs6505162 and the incidence of breast cancer. The genotyping data also showed that the CC genotype was associated with the increased risk of breast cancer in codominance (OR=2.37, 95% CI=1.29-4.35, P=0.0023, CC vs. AA), recessive (OR=2.58, 95% CI=1.48-4.52, P=0.0006; CC vs. AA+AC) and Log-additive (OR=1.43, 95% CI=1.07-1.91, P=0.016) pattern of inheritance. This indicated that the *hsa-miRw* rs6505162 C allele in comparison with the A allele could increase the risk of breast cancer (OR=1.56, 95% CI=1.13-2.16, P=0.007). Based on the AIC and BIC factors, the best fitting inheritance pattern would be a recessive model (AIC: 416.5, BIC: 424, Table 3).

Analysis of the data indicated that *hsa-miR-423* rs6505162 was associated with the tumor stage in codominance (P=0.0008, OR=4.55, 95 % CI=1.87–11.08 and OR=2.98, 95% CI=1.32-6.74 for A/C and C/C genotypes, respectively), dominance (OR=3.62, 95% CI=1.80-7.29, P=0.0002), over dominance (OR=2.87, 95% CI=1.26-6.55, P=0.0081) and Log-additive mode of inheritance (OR=1.85, 95% CI=1.21-2.84, P=0.0034). In view of the AIC and BIC factors, the best fitting model would be dominant inheritance model (AIC=187.9, BIC=194, Table 4).

Moreover, as shown in Table 5, *hsa-miR-423* rs6505162 was associated with age of the patient in a codominance (P=0.01, OR=3.01, 95% CI=1.32–6.84 and OR=2.61, 95% CI=1.18-5.78 for A/C and C/C genotypes, respectively), dominance (OR=2.79, 95% CI=1.42-5.50, P=0.0026) and Log-additive pattern (OR=1.68, 95% CI=1.12-2.51, P=0.011). The AIC and BIC indicated the presence of dominant inheritance model as the best fitting model (AIC=19909, BIC=205.9, Table 5). However, there was no association between *hsa-miR-423* rs6505162 and the estrogen and progesterone receptors in the tested inheritance models (P>0.05). Analysis of Hardy-Weinberg Equilibrium showed the presence of equilibrium for this marker in the Isfahan population (P=0.1627). Analysis of the allele frequency and heterozygosity for the indicated marker using GENEPOP website illustrated 0.356 MAF and 41.18% heterozygosity rate for *hsa-miR-423* rs6505162 (data not shown). The estimated PIC value was 0.3534 by determining the frequency of alleles per locus through PIC calculator website.

**Table 3:** Allele and genotype distribution of *hsa-miR-423* rs6505162 A>C polymorphism in the patient and control group


Model	Genotype	Control	Case	OR (95% CI)	P value	AIC	BIC

Codominant	A/A	67 (43.8)	59 (38.6)	1.00	0.0023	418	429.2
	A/C	63 (41.2)	46 (30.1)	0.83 (0.49-1.39)			
	C/C	23 (15)	48 (31.4)	2.37 (1.29-4.35)			
Dominant	A/A	67 (43.8)	59 (38.6)	1.00	0.35	427.3	434.8
	A/C-C/C	86 (56.2)	94 (61.4)	1.24 (0.79-1.96)			
Recessive	A/A-A/C	130 (85)	105 (68.6)	1.00	6e-04	416.5	424
	C/C	23 (15)	48 (31.4)	2.58 (1.48-4.52)			
Overdominant	A/A-C/C	90 (58.8)	107 (69.9)	1.00	0.042	424.1	431.5
	A/C	63 (41.2)	46 (30.1)	0.61 (0.38-0.98)			
Log-additive	---	---	---	1.43 (1.07-1.91)	0.016	422.4	429.8
Allele	A	164 (53.6)	197 (64.4)	1.00	0.007	-	-
	C	142 (46.4)	109 (35.6)	1.56 (1.13-2.16)			


Data are presented as n (%). CI; *Confidence interval,* AIC; Akaike information criterion, OR; Odds ratio, and BIC; Bayesian information criterion.

**Table 4:** Genotype and allele frequency of *hsa-miR-423 *rs6505162 A>C polymorphism among the breast cancer patient


Model	Genotype	Superficial	Invasive	OR (95% CI)	P value	AIC	BIC

Codominant	A/A	31 (58.5)	28 (28)	1.00	8e-04	189.2	198.3
	A/C	9 (17)	37 (37)	4.55 (1.87-11.08)			
	C/C	13 (24.5)	35 (35)	2.98 (1.32-6.74)			
Dominant	A/A	31 (58.5)	28 (28)	1.00	2e-04	187.9	194
	A/C-C/C	22 (41.5)	72 (72)	3.62 (1.80-7.29)			
Recessive	A/A-A/C	40 (75.5)	65 (65)	1.00	0.18	199.6	205.7
	C/C	13 (24.5)	35 (35)	1.66 (0.78-3.50)			
Overdominant	A/A-C/C	44 (83)	63 (63)	1.00	0.0081	194.4	200.5
	A/C	9 (17)	37 (37)	2.87 (1.26-6.55)			
Log-additive	---	---	---	1.85 (1.21-2.84)	0.0034	192.9	198.9


Data are presented as n (%). CI; *Confidence interval,* AIC; Akaike information criterion, OR; Odds ratio, and BIC; Bayesian information criterion.

**Table 5:** Association of *hsa-miR-423* rs6505162 with tumor stage in the breast cancer patients


Model	Genotype	>50	≤50	OR (95% CI)	P value	AIC	BIC

Codominant	A/A	32 (53.3)	27 (29)	1.00	0.01	201.8	210.9
	A/C	13 (21.7)	33 (35.5)	3.01 (1.32-6.84)			
	C/C	15 (25)	33 (35.5)	2.61 (1.18-5.78)			
Dominant	A/A	32 (53.3)	27 (29)	1.00	0.0026	199.9	205.9
	A/C-C/C	28 (46.7)	66 (71)	2.79 (1.42-5.50)			
Recessive	A/A-A/C	45 (75)	60 (64.5)	1.00	0.17	207	213.1
	C/C	15 (25)	33 (35.5)	1.65 (0.80-3.40)			
Overdominant	A/A-C/C	47 (78.3)	60 (64.5)	1.00	0.065	205.5	211.6
	A/C	13 (21.7)	33 (35.5)	1.99 (0.94-4.20)			
Log-additive	---	---	---	1.68 (1.12-2.51)	0.011	202.5	208.5


Data are presented as n (%). CI; *Confidence interval, *AIC; Akaike information criterion, OR; Odds ratio, and BIC; Bayesian information criterion.

## Discussion

In the present study, the impact of *hsa-miR-423* rs6505162 polymorphism was investigated on 306 breast cancer cases and control individuals in the Isfahan population (central province of Iran). The results indicated that *hsa-mirR-423* rs6505162 polymorphism was associated with susceptibility to increased risk of breast cancer. In this regard, recently genetic susceptibility due to SNP has been one of the major focuses of cancer molecular biology research (29).

It has been reported that MirSNPs could potentially influence miRNA maturation, silencing machinery, the structure or expression level of mature miRNA and the base pairing at the target site, altering miRNA expression. Furthermore, MirSNPs have been shown to play a functional role in miRNA-mediated gene regulation, thereby affecting susceptibility and progression of various cancers (30).

To date, a large number of investigations have been performed to unravel the exact role of MirSNPs in precursor and mature miRNA and their influences on cancer susceptibility (31, 32). *miR-423* is located in the frequently amplified region of chromosome 17q11.2 and lies within the first intron of the nuclear speckle splicing regulatory protein (*NSRP1*) gene, which is involved in alternative splicing of mRNAs. Its pre-miRNA can produce two mature transcripts, *miR-423-3p* and *miR-423- 5p,* that are at the 3´- and 5´-terminus of the pre-*miR-423*, respectively (33). The rs6505162 SNP, located in the pre-*miR-423,* 12 base pairs 5′ of *miR-423-5p* suggests a relationship with development of cancer according to cross phenotype meta-analysis (CPMA).

Up to now, most of the investigations on *miR-423* has focused on expression analyses and showed that abnormal expression of the miRNA could affect different types of cancer during cellular differentiation (34). Previous studies have shown that miRNAs (pre-miRNA SNPs) can influence the binding of nuclear factors associated with miRNA processing. Together with the other recent studies, our bioinformatics investigation suggested that rs6505162 might influence the expression of miR-423 (data not shown). However, some experiments assessing the rs6505162 SNP effect on function of miR-423 are needed.

Moreover, the SNP of rs895819 in pre-*miR-27a* has been reported as a risk factor for breast cancer in younger Chinese populations (35). Experiments on rs6505162 polymorphism and risk of cancer have yielded unpredictable results. Ye et al. (36) reported that C allele of rs6505162 was significantly higher in the cancer patients compared to the controls in 346 Caucasian ESCC patients. Another study showed that the C genotype of rs6505162 SNP decreases breast cancer development risk. Nevertheless, one study showed that the C genotype of rs6505162 suggested an increased risk of developing breast and ovarian cancers in carriers with Breast Cancer Associated 2 (BRCA2) mutation (34).

Genotyping data obtained from this study showed that *hsa-miR-423* rs6505162 could have a positive effect on the incidence of breast cancer with approximately 2.5 folds. This suggested that *hsa-miR-423* rs6505162 could involve in the susceptibility to increase risk of breast cancer in the Isfahan population. Zhao et al. (19) reported that *miR-423* could play potentially an oncogenic role in breast tumorigenesis. Moreover, it has been reported that this MirSNP showed several associations with cancer development in diverse ethnicities (34). One example includes the study performed by Kontorovich et al. (33) and Hu et al. (34), whereby it was illustrated that rs6505162 was associated with a significantly increased risk of ovarian and bladder cancers.

On the other hand, another study showed that *miR- 423* could confer a reduced risk of breast and esophageal cancers as well as the recurrence or survival of renal cell carcinoma and prostate cancer (36). Interestingly, we found the association between clinicopathological characteristics and *hsa-miR-423* rs6505162 polymorphism in breast cancer patients. The association analysis indicated that existence of C recessive (minor) allele in patients who were >50 years old had 2-3 folds higher risk of developing breast cancer. Moreover, 2-4.5 folds increase in the risk of developing breast cancer was observed in those with invasive stages of breast cancer progression compared to those at the superficial stages.

Given the association of *hsa-miR-423* rs6505162 polymorphism with increased risk of breast cancer, its allele frequency in control population was investigated. The data showed presence of Hardy-Weinberg equilibrium for *hsa-miR-423* rs6505162 marker in the Isfahan population. The data indicated a high heterozygosity for *hsa-miR-423* rs6505162 variant (with He=41.18%). Moreover, analysis showed PIC=0.3534. This indicated diversity of hsamiR- 423 rs6505162 in the Isfahan population. In the present study, MAF was compared to the other population based on the 1000 genome project database (data not shown). Data showed that the highest MAF was related to Chinese population (0.8127) indicating a big difference with the Iranian population. The lowest MAF refers to the American population (0.2541).

On the other hand, the British population has the closest distance to the Iranian population for both A and C alleles of *hsa-miR-423* rs6505162 polymorphism. All together, the data from this study showed that there was an association between *hsa-miR-423* rs6505162 A/C polymorphism and susceptibility to breast cancer in the population of Isfahan central province of Iran. We highly suggest using anti-sense RNA of *hsa-miR-423* rs6505162 in animal model to evaluate this point.

## Conclusion

Data obtained from this study could suggest *hsa-miR-423* rs6505162 as a new molecular marker in molecular cancer diagnosis and prevention as well as the development of possible individually tailored miRNA-based therapy. Furthermore, these data may indicate that polymorphism in pre-microRNA (*hsa-miR-423* rs6505162) could play a role in the pathogenesis of breast cancer.
